# Social Memory Formation Rapidly and Differentially Affects the Motivation and Performance of Vocal Communication Signals in the Bengalese Finch (*Lonchura striata* var. *domestica*)

**DOI:** 10.3389/fnbeh.2016.00113

**Published:** 2016-06-14

**Authors:** Danielle C. Toccalino, Herie Sun, Jon T. Sakata

**Affiliations:** ^1^Integrated Program in Neuroscience, McGill UniversityMontreal, QC, Canada; ^2^Department of Biology, McGill UniversityMontreal, QC, Canada; ^3^Center for Research in Behavioral NeurobiologyMontreal, QC, Canada

**Keywords:** directed song, birdsong, courtship, vocal performance, songbird

## Abstract

Cognitive processes like the formation of social memories can shape the nature of social interactions between conspecifics. Male songbirds use vocal signals during courtship interactions with females, but the degree to which social memory and familiarity influences the likelihood and structure of male courtship song remains largely unknown. Using a habituation-dishabituation paradigm, we found that a single, brief (<30 s) exposure to a female led to the formation of a short-term memory for that female: adult male Bengalese finches were significantly less likely to produce courtship song to an individual female when re-exposed to her 5 min later (i.e., habituation). Familiarity also rapidly decreased the duration of courtship songs but did not affect other measures of song performance (e.g., song tempo and the stereotypy of syllable structure and sequencing). Consistent with a contribution of social memory to the decrease in courtship song with repeated exposures to the same female, the likelihood that male Bengalese finches produced courtship song increased when they were exposed to a different female (i.e., dishabituation). Three consecutive exposures to individual females also led to the formation of a longer-term memory that persisted over days. Specifically, when courtship song production was assessed 2 days after initial exposures to females, males produced fewer and shorter courtship songs to familiar females than to unfamiliar females. Measures of song performance, however, were not different between courtship songs produced to familiar and unfamiliar females. The formation of a longer-term memory for individual females seemed to require at least three exposures because males did not differentially produce courtship song to unfamiliar females and females that they had been exposed to only once or twice. Taken together, these data indicate that brief exposures to individual females led to the rapid formation and persistence of social memories and support the existence of distinct mechanisms underlying the motivation to produce and the performance of courtship song.

## Introduction

The expression of social behaviors is modulated by cognitive processes. In particular, social memory and familiarity can influence the expression of various forms of social behavior, including reproductive (Miller, 1979; O’Loghlen and Beecher, 1999; Ophir and Galef, 2004; Vignal et al., 2008; Griffith and Ejima, 2009; Iwasaki et al., 2013; Heinig et al., 2014), parental (McCabe and Horn, 1994; Horn, 2004; Town and McCabe, 2011), and aggressive behaviors (Stoddard et al., 1991; Temeles, 1994; Bradbury and Vehrencamp, 2011; Nishizawa et al., 2011; Grabowska-Zhang et al., 2012; Jaška et al., 2015). For example, in both mice and rats, the amount of time an individual spends investigating or vocalizing in response to another individual decreases as a function of familiarity (D’Amato, 1997; D’Amato and Moles, 2001; Moles et al., 2007; Musolf et al., 2010; Lukas et al., 2013; Perna et al., 2015). In socially monogamous voles, mating leads to the formation of a social memory for the mate which then affects mating, affiliative, and aggressive behaviors toward conspecifics (Young and Wang, 2004; Young et al., 2008; Blocker and Ophir, 2015). Additionally, across various species, females preferentially mate with males that they have observed to win aggressive interactions (Mennill et al., 2002; Ophir and Galef, 2004; White, 2004; Bradbury and Vehrencamp, 2011) or observed to have mated with other females (“mate copying”: Schlupp et al., 1994; Valone and Templeton, 2002; Galef, 2008). As such, understanding the processes underlying memory formation and individual recognition are important for understanding mechanisms underlying the expression of social behaviors (Insel and Fernald, 2004).

While the influence of social memory on the likelihood of social behavioral expression is well established, we know relatively little about the degree to which distinct components of social behavior differentially reflect social memory or about the factors that influence the rapidity and persistence of social memory formation. For example, many studies have found that social memory formation affects the motivation to copulate with individual females (e.g., Wilson et al., 1963; Dewsbury, 1981; Johnston and Rasmussen, 1984; Tan et al., 2013; Schnell et al., 2014) but only a handful of studies have examined how familiarity could affect the performance of copulatory behaviors (e.g., Dewsbury, 1979; Riters and Balthazart, 1998). Males of many vertebrate species produce courtship signals to solicit copulation from females, and social memory could differentially affect the motivation and performance of courtship behaviors. For example, male Barbary doves produce more bowing displays when courting unfamiliar females than when courting familiar partners (Erickson and Morris, 1972), and male zebra finches produce more courtship songs to unfamiliar females (Caryl, 1976). However, the degree to which familiarity affects the stereotypy and intensity of such displays remains largely unknown.

Additionally, relatively little is known about how the amount of social interactions influences the rapidity and persistence of social memory formation. A number of studies have found that a single exposure to a conspecific can influence the nature of subsequent interactions with familiar individuals (e.g., Hilliard et al., 1997; D’Amato and Moles, 2001; Moles et al., 2007; Lukas et al., 2013; Perna et al., 2015). However, few studies directly examine how different amounts of social interactions affect distinct aspects of social behavior or the persistence of behavioral changes (e.g., Guan and Dluzen, 1994; Sakata et al., 2002). Understanding the degree to which responses to familiar individuals persist over hours to days is important because such persistence could reflect the longer-term consolidation of social memories.

Here we conducted a series of experiments to reveal how social memory formation affects the production and performance of courtship song in male Bengalese finches. Songbirds like the Bengalese finch have been critical for understanding mechanisms underlying the learning and control of social and communicative behaviors (Dong and Clayton, 2009; Bolhuis et al., 2010; Sakata and Vehrencamp, 2012; Brainard and Doupe, 2013; Bertram et al., 2014; Woolley and Kao, 2015). Male Bengalese finches are excellent for revealing the degree to which social memory formation differentially affects the motivation to produce and the performance of courtship song because they produce courtship songs to solicit copulations from females and alter various aspects of their song performance during courtship interactions (Sakata and Vehrencamp, 2012; Dunning et al., 2014; James and Sakata, 2015; Matheson et al., 2016). We hypothesized that exposures of males to females would rapidly affect the display of male courtship song, that the magnitude of the effect of familiarity would increase with more exposures to females, and that familiarity could differentially affect the motivation to produce and the performance of male courtship song.

## Materials and Methods

### Animals

Adult male Bengalese finches (>4 months; *n* = 16) were raised in our colony at McGill University or purchased from vendors (Exotic Wings and Pet Things, Ontario, ON, Canada). Outside of experimental periods, males were housed in same-sex group cages and not able to see or hear stimulus females. Birds were housed on a 14L:10D light cycle and provided food and water* ad libitum*. All procedures were approved by the McGill University Animal Care and Use Committee and performed in accordance with guidelines of the Canadian Council on Animal Care.

### Behavioral Testing

#### General

Beginning at least one day before experimentation, male Bengalese finches were housed individually in cages (20 × 20 × 20 cm) in sound-attenuating chambers (“soundboxes”; TRA Acoustics, Cornwall, Ontario, ON, Canada). For all experiments, we analyzed the production of courtship song by briefly exposing male Bengalese finches to individual females (Sakata et al., 2008; Sakata and Brainard, 2009; James and Sakata, 2015; Matheson et al., 2016). In brief, during experimental sessions, we exposed individual male Bengalese finches to a female housed in a separate cage and assessed whether he produced courtship song. The courtship songs of Bengalese finches are readily distinguishable from non-courtship songs and are generally associated with a male facing or approaching a female, performing a courtship dance (e.g., pivoting body from side to side), and fluffing his plumage (Morris, 1954; Zann, 1996). Behavior was remotely monitored by video camera, and only songs that were accompanied by at least two of the above behaviors were categorized as courtship songs. Male Bengalese finches produce courtship songs within seconds of the introduction of a stimulus female and can produce multiple courtship song bouts in rapid succession during each female presentation. For all experiments, females were removed after the termination of a male’s courtship song or after ~30 s if the male did not sing, and the interval between exposures to females was 4–5 min. Non-courtship song bouts are spontaneously produced when birds are alone and generally separated by seconds to minutes. Behavioral tests were conducted 2–6 h after lights came on.

All males were first screened for courtship vigor by exposing them to six different stimulus females. Only males that courted at least 50% of the females during these screening tests were included in experiments (*n* = 16 males).

#### Effects of Familiarity on the Production and Performance of Courtship Song

**Figure 1:**
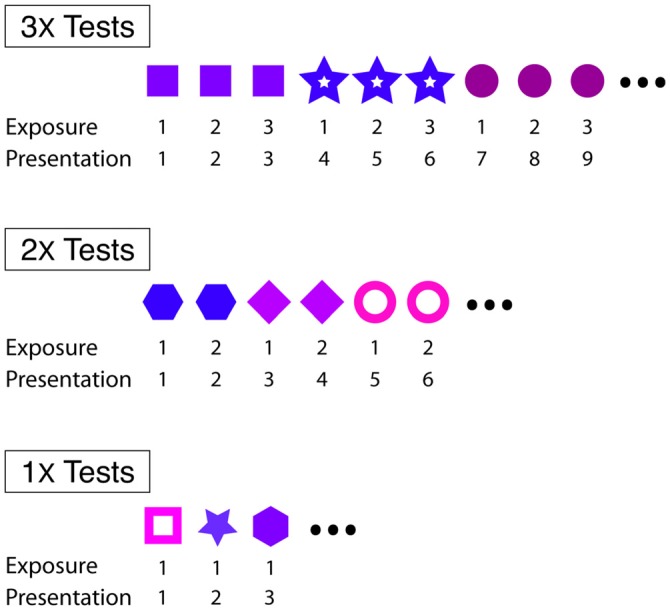
**Experimental designs for 3X, 2X and 1X tests.** For all tests, males were briefly (<30 s) exposed to individual females at 4–5 min intervals. Within each type of test, different symbols refer to different females.

We investigated short- and longer-term effects of familiarity on courtship song performance. In general, we assessed the effect of familiarity on courtship song performance by consecutively exposing male Bengalese finches to individual females 1–3 times. For “3X tests” (*n* = 16) and “2X tests” (*n* = 10), males were exposed to individual females three or two consecutive times, respectively, before changing the identity of the stimulus female (Figure 1). For “1X tests” (*n* = 13), male Bengalese finches were exposed to an individual female on only one occasion before changing the identity of the stimulus female (Figure 1). Males were exposed, on average, to 7.9 (range: 6–10) and 12.1 (range: 11–15) different stimulus females during 3X and 1X tests, respectively. All males were exposed to eight different stimulus females during 2X tests. We revealed short-term effects of familiarity by analyzing changes to the likelihood and acoustic structure of courtship song across consecutive exposures to stimulus females.

We also investigated the degree to which memories for individual females persisted over days and the effect of repeated female exposures on the formation of longer-term social memories. To this end, we analyzed the degree to which a random subset of the males given 3X (*n* = 13), 2X (*n* = 10), and 1X (*n* = 7) tests produced courtship song to familiar and unfamiliar females 2 days after 3X, 2X, and 1X tests. For these tests of longer-term memory (“longer-term memory tests”), we presented each experimental male with stimulus females that he had been exposed to 2 days earlier (“familiar females”) and with females that he had not been exposed to 2 days earlier (“unfamiliar females”). We used the testing protocol described above for 1X tests and exposed experimental males to individual stimulus females only once before changing the identity of the female. The order of female presentations was pseudo-randomized such that familiar and unfamiliar females were presented at comparable periods in the testing session (e.g., across all memory tests, median rank order for familiar and unfamiliar females was not different: *F*_(1,29)_ = 0.1; *p* = 0.7935). During longer-term memory tests following 3X and 1X tests, males were exposed, on average, to 16.0 (range: 12–20) and 18.6 (range: 12–24) stimulus females, respectively. During longer-term memory tests following 2X tests, all males were exposed to 16 stimulus females. Experimental males were exposed to the same number of familiar and unfamiliar females during longer-term memory tests for all but 2 out of 30 cases (in the outlying cases, males were exposed to 10–20% more novel females than familiar females).

All 16 males were administered 3X tests. In addition, random subsets of males were also administered 1X (*n* = 6), 2X (*n* = 3), or both 1X and 2X tests (*n* = 7) in conjunction with 3X tests. For those birds that were administered 3X and 1X tests (*n* = 13), the order of testing was randomized (five underwent 3X tests first, eight underwent 1X tests first). 2X tests were conducted in response to results from 3X and 1X tests and followed 3X and 1X tests. Tests were separated by a minimum of 2 months.

### Song Collection and Analysis

For all tests, birds were housed individually in soundboxes, and song was recorded using an omnidirectional microphone (Countryman Associates, Inc., Menlo Park, CA, USA) positioned above the male’s cage. A computerized, song-activated recording system was used to detect and digitize song (Sound Analysis Pro v.1.04: http://ofer.sci.ccny.cuny.edu/html/sound_analysis.html, digitized at 44.1 kHz). Recorded songs were digitally filtered (0.3–8 kHz) for off-line analysis using software custom-written in the Matlab programming language (The MathWorks, Natick, MA, USA).

We analyzed the courtship and non-courtship songs produced by male Bengalese finches during 3X tests to examine the effects of social context and familiarity on song performance. We analyzed the effect of social context by comparing courtship and non-courtship songs (e.g., Kao and Brainard, 2006; Sakata et al., 2008; Woolley et al., 2014; James and Sakata, 2015). Males were exposed to females at 4–5 min intervals, which allowed for the collection of non-courtship song between female exposures. However, not all birds produced renditions of non-courtship song between female presentations; therefore, non-courtship songs were also recorded for 30 min before and after testing sessions (e.g., Sakata et al., 2008; Sakata and Brainard, 2009; James and Sakata, 2015). To increase the reliability of our estimates of means and variances for each condition, we analyzed the effect of social context on song performance only in males that produced courtship song on more than three presentations of females and produced more than three non-courtship songs during the experimental period (*n* = 14 males).

We analyzed the effect of familiarity on song performance in two ways. First, we compared the structure of courtship songs produced during a male’s first exposure to stimulus females to the structure of courtship songs produced during all subsequent exposures to those same females during 3X tests. To increase the reliability of our estimates of means and variances for each condition, we only analyzed songs from males that produced at least three songs during their first exposure to females and at least three songs during all subsequent exposures to females (*n* = 12 males). Second, we compared the acoustic structure of courtship songs produced to familiar and to unfamiliar females during longer-term memory tests that followed 3X tests. We analyzed only the songs of males that produced courtship song to at least three familiar and at least three unfamiliar females during these tests (*n* = 7 males). For both of these analyses, we were particularly interested in testing the hypothesis that courtship songs become less distinct from non-courtship songs as females become more familiar.

We analyzed a variety of song features that are affected by social interactions and are salient to females, including syllable sequencing, song tempo, syllable structure, and song durations (Kao and Brainard, 2006; Sakata et al., 2008; Woolley and Doupe, 2008; Hampton et al., 2009; Sakata and Brainard, 2009; Dunning et al., 2014; Heinig et al., 2014; James and Sakata, 2015; Matheson et al., 2016). Bengalese finches produce bouts of song that consist of distinct acoustic elements (“syllables”) arranged in both stereotyped and variable sequences. “Syllables” are individual acoustic elements that are separated from each other by >5 ms of silence, and “song bouts” are contiguous segments of song that are separated from each other by >500 ms of silence (Sakata et al., 2008). Identical to previous studies, we manually labeled syllables based on visual inspection of spectrograms following amplitude-based syllable segmentation in Matlab (e.g., Sakata et al., 2008; Heinig et al., 2014; James and Sakata, 2014; Matheson et al., 2016). Song was labeled by two experts (HS and JTS) who were blind to the social context and the familiarity of stimulus females.

Bengalese finch song is characterized by nodes with variable sequencing called “branch points”, and we analyzed changes to syllable sequencing at such branch points. Sequence variability at branch points is not simply biological noise but reflects a controlled aspect of song that is stable over days and weeks and modulated by social context (Okanoya and Yamaguchi, 1997; Sakata et al., 2008; Hampton et al., 2009; Warren et al., 2012; Heinig et al., 2014; James and Sakata, 2014, 2015; Matheson and Sakata, 2015a). We analyzed the probability of different syllable transitions at branch points (typically 2–5 distinct transitions per branch point), paying close attention to longer-range statistics in sequencing (Fujimoto et al., 2011; Warren et al., 2012; James and Sakata, 2014; Matheson et al., 2016). Stereotyped and branch point sequences were visually selected during manual labeling and confirmed using bigram plots (e.g., Okanoya and Yamaguchi, 1997; Kakishita et al., 2008; Fujimoto et al., 2011; Matheson et al., 2016). Sequences were considered to be branch points if the transition probability for any transition from that sequence was <95% under any of the conditions being compared. We computed the transition entropy, a measure of variability, of each branch point using the following formula:

$$\text{transition entropy}=\Sigma -p_{i}*\log_{2}p_{i}$$

where, the sum is over all transitions produced at the branch point, and *p*_i_ is the probability of the ith transition across all renditions of the branch point. Transition entropy summarizes the variability of syllable sequencing: branch points with more variable and unpredictable transitions (i.e., closer to uniform probability) have higher transition entropy scores. Only branch points that occurred at least 10 times within each condition were included in the analysis. Instances in which song was terminated immediately following the branch point were not included in the calculation of entropy.

Changes to song tempo were analyzed using methods similar to previous studies (Cooper et al., 2012; James and Sakata, 2014, 2015). Specifically, we identified and measured the duration of a single, commonly produced sequence in an individual’s song. Sequence durations were defined as the interval between the onset of the first syllable of the sequence to the onset of the last syllable of the sequence. Onsets were used to compute sequence durations because onsets are sharper and more consistent across renditions, leading to a more stable and reliable estimate of song tempo. Because sequence durations increase as the song progresses (Chi and Margoliash, 2001; Cooper and Goller, 2006; Glaze and Troyer, 2006) and because song durations can change as a function of social context and familiarity, we restricted our analysis to the first occurrence of the sequence in each song (e.g., James and Sakata, 2014, 2015; Matheson et al., 2016).

To analyze changes to syllable structure, we calculated the fundamental frequency (FF) of syllables that had distinct and stable harmonic structure. The FF of such syllables is tightly regulated by the nervous system and represents an important metric of song performance (Mooney, 2009; Sakata and Vehrencamp, 2012; Brainard and Doupe, 2013). To compute the FF, we calculated the autocorrelation of a segment of the sound waveform (8- or 16-ms window) and defined the FF as the distance, in Hz, between the zero-offset peak and the highest peak in the autocorrelation function. To improve the resolution and accuracy of frequency estimates, we performed a parabolic interpolation of the peak of the autocorrelation function (de Cheveigné and Kawahara, 2002). Each rendition of a syllable was visually screened to ensure that we analyzed only examples devoid of sound artifacts that could affect FF calculations (e.g., sound of movement, female calls in background). Only syllables that occurred more than 10 times within each condition were included in the analysis. For each syllable, we computed the mean and variability of FF across renditions, two aspects that have been found to change across social contexts (e.g., Sakata et al., 2008; Hampton et al., 2009). We characterized the variability of FF across renditions using the coefficient of variation (CV: standard deviation/mean).

In addition to these features, we also analyzed context- and familiarity-dependent changes to the number of introductory notes preceding song onset and to song duration. Introductory notes are brief, low amplitude vocal elements with simple acoustic structure that are repeated consecutively before song onset. We counted introductory notes beginning with the first introductory note preceding the first song syllable, counting backwards until we reached >500 ms of silence (Sakata et al., 2008; Hampton et al., 2009; Matheson and Sakata, 2015a). We defined the duration of a song bout as the interval from the onset of the first syllable of the song bout to the offset of the last syllable of the song bout. Because males can rapidly produce multiple song bouts during a single presentation of a female, we calculated courtship song durations as the sum duration of all courtship song bouts produced during each female presentation.

### Statistical Analyses

We analyzed the effect of familiarity on the production and performance of courtship song. To examine the effect of familiarity on the motivation to produce courtship song, we analyzed the proportion of presentations in which males produced courtship song. To analyze the short-term effects of familiarity on courtship song production, we analyzed the proportion of presentations during which males produced courtship song as a function of exposure number (e.g., 1st, 2nd, or 3rd exposures to individual stimulus females during 3X tests). To analyze longer-term effects of familiarity on the motivation to produce courtship song, we compared the proportion of presentations of familiar or unfamiliar females during which males produced courtship song. In addition to analyzing the effects of familiarity on the motivation to produce song, we also analyzed short- and longer-term effects of familiarity on measures of song performance (transition entropy of branch points, sequence durations, mean and CV of FF, the number of introductory notes preceding song, and song durations) as well as context-dependent changes to these features.

All data were analyzed using mixed effects models. For example, in our analysis of changes to the likelihood of courtship song across consecutive exposures to females, the proportion of presentations in which a male produced courtship song was the dependent variable, exposure number (1st, 2nd, or 3rd) was the independent variable, and bird ID was a random variable (repeated-measures analysis; Gotelli and Ellison, 2013). We confirmed that sphericity was not violated before running these models (Mauchly criterion, α = 0.05). Similar mixed effects models were used to assess differences in the likelihood of courtship song production during tests for longer-term memory (e.g., 2 days after 3X tests) as well as differences in measures of song performance between non-courtship and courtship songs, between courtship songs produced during the first and subsequent exposures to females, and between courtship songs produced to familiar and unfamiliar females. Because we measured sequence variability (transition entropy) at multiple branch points and the FF of multiple syllables per bird, we also included branch point ID nested within bird ID or syllable ID nested within bird ID as a random variable for the analyses of syllable sequencing and structure. Tukey’s HSD was used for all *post hoc* contrasts.

## Results

### Effects of Repeated Exposures to Females on the Motivation to Produce and the Performance of Courtship Song

**Figure 2:**
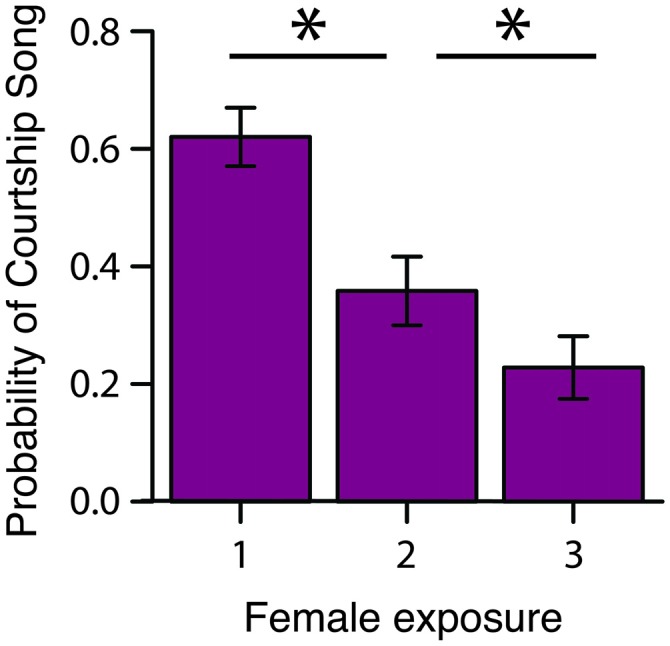
**Familiarity rapidly affects the motivation to produce courtship song.** During 3X tests, the probability that a male produced courtship song (i.e., proportion of presentations in which a male produced courtship song) significantly decreased from the 1st to 2nd and 2nd to 3rd exposure to a female (*n* = 16 males). *p < 0.05.

To assess how familiarity affects the motivation to produce courtship song, we briefly exposed male Bengalese finches to a stimulus female on three consecutive occasions before presenting the male with a different stimulus female (“3X tests”; *n* = 16 males; Figure 1). We computed the proportion of presentations in which a male produced courtship song to a female as a function of the number of exposures to the female (Table 1). We observed that the proportion of presentations in which a male produced courtship song significantly decreased with repeated exposures to an individual female (*F*_(2,30)_ = 6.1, *p* < 0.0001; Figure 2). Males produced courtship song on 60%, 34%, and 23% of the 1st, 2nd, and 3rd exposures to individual females, respectively (Table 1). All contrasts were significant (Tukey’s HSD, *p* < 0.05), indicating that the likelihood that a male would produce courtship song significantly decreased from the first to second, the first to third, and the second to third exposure.

**Table 1:** **Number of stimulus females and number of presentations with courtship song for each male administered 3X tests (i.e., male exposed to stimulus female on three consecutive occasions before switching the stimulus female)**.

		FIRST EXPOSURE		SECOND EXPOSURE		THIRD EXPOSURE	
Male ID	# of females	# of presentations with courtship song	proportion of presentations with courtship song	# of presentations with courtship song	proportion of presentations with courtship song	# of presentations with courtship song	proportion of presentations with courtship song
o174br12	6	4	0.67	4	0.67	3	0.50
s935	6	4	0.67	3	0.50	1	0.17
s2295	7	5	0.71	5	0.71	3	0.43
bl12w13	8	4	0.50	4	0.50	6	0.75
black	8	5	0.63	3	0.38	1	0.13
o189br53	8	6	0.75	1	0.13	2	0.25
s1408g	8	5	0.63	2	0.25	1	0.13
p163y48	8	3	0.38	0	0.00	0	0.00
p173y53	8	7	0.88	3	0.38	2	0.25
p183y25	8	4	0.50	1	0.13	0	0.00
bl27p163	8	3	0.38	2	0.25	1	0.13
bl93p188	8	5	0.63	3	0.38	0	0.00
p177y27	8	2	0.25	0	0.00	0	0.00
w34o156	8	8	1.00	5	0.63	5	0.63
o138br52	10	4	0.40	1	0.10	1	0.10
s936	10	6	0.60	5	0.50	2	0.20

**Figure 3:**
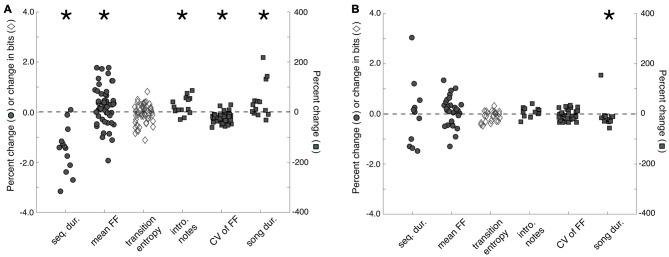
**Changes to song structure as a function of social context and familiarity. (A)** Sequence durations (“seq. dur.”) were significantly shorter, song durations (“song dur.”) were significantly longer, mean fundamental frequency (FF; “mean FF”) and the number of introductory notes (“intro. notes”) produced before song were significantly higher, and the variability of FF (“CV of FF”) was significantly lower for courtship songs than for non-courtship songs. To facilitate data visualization and feature comparisons, we present the percent changes (from non-courtship song to courtship song) for all features except transition entropy. For transition entropy, the change (in bits) from non-courtship song to courtship song is plotted. **(B)** To assess the effect of familiarity on song structure, we compared the structure of courtship songs produced during the first exposure to a female (i.e., when males are more likely to produce courtship song to a female) to the structure of courtship songs produced during subsequent exposures to a female (i.e., when males are less like to produce courtship song to a female). Only courtship song duration significantly decreased from the first exposure to subsequent exposures. Plotted is the change in transition entropy and the percent changes for all other features from courtship songs produced on a male’s first exposure to individual females to courtship songs produced on subsequent exposures to females. For both **(A,B)**, filled circles and open diamonds scale to the left *y*-axis, whereas filled squares scale to the right *y*-axis. Introductory notes and sequence and song durations were analyzed per bird, whereas the mean and CV of FF and transition entropy were analyzed per syllable or sequence (see “Results” Section). “*” Indicates *p* < 0.05.

Memory for individual females may also be reflected in measures of song performance. Before analyzing the effect of familiarity on song performance, we first confirmed that exposures to females changed song performance. We compared the songs that males produced to females during courtship interactions (“courtship songs”) and the songs that males produce when alone (“non-courtship songs”) during 3X tests. We analyzed the songs of males that produced courtship songs on at least three presentations to stimulus females (*n* = 14 males) and analyzed, on average, 11.9 ± 1.3 (mean ± SEM; range: 5–20) courtship songs and 19.4 ± 1.9 (range: 12–35) non-courtship songs per male. Overall, we observed context-dependent changes to song that were similar to previously documented changes (e.g., Kao and Brainard, 2006; Sakata et al., 2008; Matheson et al., 2016; Figure 3A). Specifically, sequence durations were significantly shorter during courtship song than during non-courtship song (*F*_(1,13)_ = 9.3, *p* = 0.0092), indicating that courtship song was faster than non-courtship song. Courtship songs were preceded by significantly more introductory notes (*F*_(1,13)_ = 5.1, *p* = 0.0412) and were longer in duration (*F*_(1,13)_ = 4.7, *p* = 0.0483) than non-courtship songs. Additionally, the FF of syllables with flat, harmonic structure (*n* = 51 syllables) was significantly higher (*F*_(1,50)_ = 7.5, *p* = 0.0087) and less variable (*F*_(1,50)_ = 65.0, *p* < 0.0001) when males produced courtship songs. Whereas previous studies have documented that syllable sequencing at branch points is less variable during courtship song than non-courtship song (e.g., Sakata et al., 2008; Sakata and Brainard, 2009; Heinig et al., 2014; James and Sakata, 2015; Matheson and Sakata, 2015b; Matheson et al., 2016), transition entropy (*n* = 50 branch points) was not significantly different between the courtship and non-courtship songs of males in this study (*F*_(1,49)_ = 1.4, *p* = 0.2388).

To assess the degree to which these measures of song performance can serve as indices of social memory, we compared the structure of courtship songs that males produced on their first exposure to individual females to courtship songs that males produced on their subsequent exposures to the same females. Because the likelihood that a male produced courtship song to an individual female significantly decreased following the first exposure, we combined the songs that each male produced on his second and third exposures to females and compared these songs to the courtship songs produced on first exposures. This allowed us to compare datasets with similar numbers of songs (*p* = 0.5208). We analyzed the songs of males that produced at least three songs during their first exposure to females and at least three songs during all subsequent exposures to females (*n* = 12 males) and examined, for each male, 6.9 ± 0.8 (range: 4–14) courtship songs produced on first exposures and 6.0 ± 0.9 (range 3–10) courtship songs produced on subsequent exposures.

We found that courtship song durations on the first exposure to a female (i.e., when a male is more likely to produce courtship song to a female) were significantly longer than courtship song durations on subsequent exposures to females (i.e., when a male is less likely to produce courtship song to a female; *F*_(1,11)_ = 5.1, *p* = 0.0444; Figure 3B). However, the number of introductory notes preceding song, song tempo, the mean and variability of FF (*n* = 30 syllables), and the stereotypy of syllable sequencing (*n* = 23 branch points) were not significantly different between courtship songs produced on first and subsequent exposures to stimulus females (*p* > 0.05 for all; Figure 3B).

### Disentangling the Effects of Social Memory and “Courtship Fatigue”

The decrease in the production of courtship song with repeated female presentations could result from fatigue on the part of the male rather than the formation of social memories. To assess the degree to which such “courtship fatigue” contributed to the decrease in courtship song during 3X tests, we conducted a series of additional analyses and experiments. First, we analyzed how the likelihood of courtship song changed when we presented males with a different female after three repeated exposures to an individual female. If fatigue was the primary reason for decreases in the likelihood of courtship song during 3X tests, we would expect similar decreases when males were exposed to an unfamiliar female as when males were exposed to the same female on consecutive presentations. In contrast, if decreases in courtship song production were due to the formation of social memories, we would expect the likelihood of courtship song to *decrease* when males were consecutively exposed to the same female (i.e., habituation) and to *increase* when males were exposed to a different female for the first time (i.e., dishabituation).

**Figure 4:**
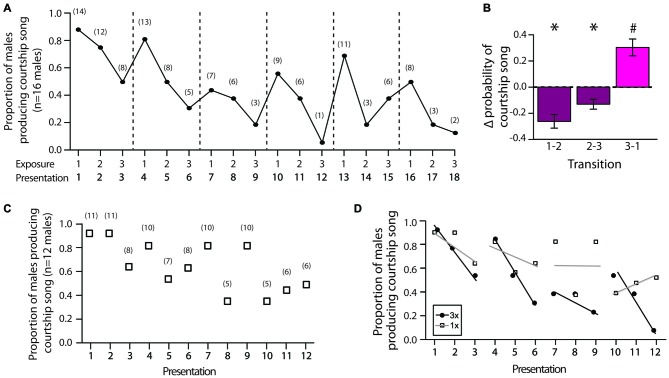
**Decreases in the production of courtship song reflect the formation of short-term memories. (A)** Plotted are the proportion of male Bengalese finches (*n* = 16) that produced courtship song across each exposure to the first six stimulus females during 3X tests (range: 6–10 individual females). Dashed lines indicate when the identity of the stimulus female was changed. Numbers in parentheses indicate the number of males producing courtship song for each presentation. **(B)** The proportion of presentations in which males produced courtship song changed in a systematic manner across exposures (3X tests). Plotted is the mean *change* (±SEM) in the likelihood of courtship song (i.e., change in the proportion of presentations that an individual male produced courtship song) from the first to second exposure to a female (“1–2”), from the second to third exposure to a female (“2–3”), and from the third exposure to a female to the first exposure to a different female (“3–1”). “*” Indicates significantly less than zero (*p* < 0.05), and “#” indicates significantly greater than zero (*p* < 0.05) and different than “1–2” and “2–3”. **(C)** The proportion of males producing courtship song to individual females gradually decreased over presentations even when the identity of the female was changed after a single exposure (1X tests; 12 stimulus females). Numbers in parentheses indicate the number of males producing courtship song for each presentation. **(D)** We compared the rates of change in the proportion of males producing courtship song during 3X tests, in which males were consecutively exposed to the same female (presentations 1–3, 4–6, 7–9, and 10–12; black lines), to the rates of change across the same presentations during 1X tests, in which males were consecutively exposed to different females (gray lines). We found that the proportion of males producing courtship song decreased at a faster rate when males were exposed to the same female across presentations than when they were exposed to different females across presentations. For this analysis, only data for males that were administered both 1X and 3X tests were examined (*n* = 13 males).

We first analyzed changes in the proportion of males that produced courtship song across female exposures during 3X tests. Consistent with the notion that social memory contributes to changes in courtship song production, we found that the proportion of males producing courtship song generally decreased when males were exposed to the same female across consecutive presentations (i.e., from the first to second exposure and from the second to third exposure; Figure 4A). In addition, we observed that the proportion of males producing courtship song generally increased when males were exposed to a different female for the first time (Figure 4A). To analyze the significance of these changes, for each male we computed the *change* in the proportion of presentations with courtship song (i.e., the change in the likelihood that a male produced courtship song) from the first to second exposure to an individual female (“1–2”), from the second to third exposure to an individual female (“2–3”), and from the third exposure to a female to the first exposure to a different female (“3–1”). Changes in the likelihood of courtship song across these consecutive exposures were significantly different (*F*_(2,30)_ = 21.9, *p* < 0.0001; Figure 4B). Specifically, the change in the likelihood of courtship song when males were exposed to a different female (i.e., from the third exposure to a female to the first exposure to a different female) was significantly more positive than the changes in the likelihood of courtship song when males were consecutively exposed to the same female (i.e., from the first to second exposure and from the second to third exposure to an individual female; Tukey’s HSD, *p* < 0.05 for both contrasts; Figure 4B). Furthermore, whereas changes to likelihood of courtship song were significantly negative when the female remained the same (*t*-test; H_0_: mean = 0; *p* < 0.05 for both), the change in the likelihood of courtship song was significantly positive when the stimulus female was changed (*p* = 0.0028; Figure 4B).

The previous analysis does not rule out the possibility that fatigue could contribute to some extent to changes in courtship song production over the testing session. Indeed, the proportion of males that produced courtship song on their first exposure to a female tended to decrease over the course of the entire testing session, suggesting that fatigue can affect the likelihood of courtship song over the testing period. To further assess the potential contribution of fatigue, we investigated how the proportion of males producing courtship song changed when we switched the stimulus female after single exposures (“1X tests”; *n* = 13 males). There was generally a linear decrease in the proportion of males producing courtship song across the 12 presentations of stimulus females (*F*_(1,10)_ = 10.3, *p* = 0.0093; slope: −3.7 ± 1.1%; Figure 4C).

To quantify the relative contributions of social memory and fatigue to courtship song production, we compared the rates of change in the proportion of males producing courtship song across presentations between 3X and 1X tests. All of the males given 1X tests were also administered 3X tests, allowing us to directly compare the rates of change in a paired manner. For both 3X and 1X tests, there was a significant negative relationship between exposure number and the proportion of males producing courtship song (*p* < 0.01 for both). However, the rate of change was steeper during 3X tests (slope ± SEM: −5.6 ± 1.3%) than during 1X tests (slope ± SEM: −3.7 ± 1.1%). This difference is even more striking when examining the rate of change across presentations in which females remained the same for 3X tests (i.e., presentations 1–3, 4–6, 7–9, and 10–12). The average rate of change when males were exposed to the same female during 3X tests was −19.2 ± 4.0%. In contrast, the average rate of change was only −3.4 ± 4.0% for 1X tests over the same ranges of presentations, and this difference between 3X and 1X tests approached significance (*F*_(1,3)_ = 9.6, *p* = 0.0531; Figure 4D).

### Persistence of Social Memories Across Days

**Figure 5:**
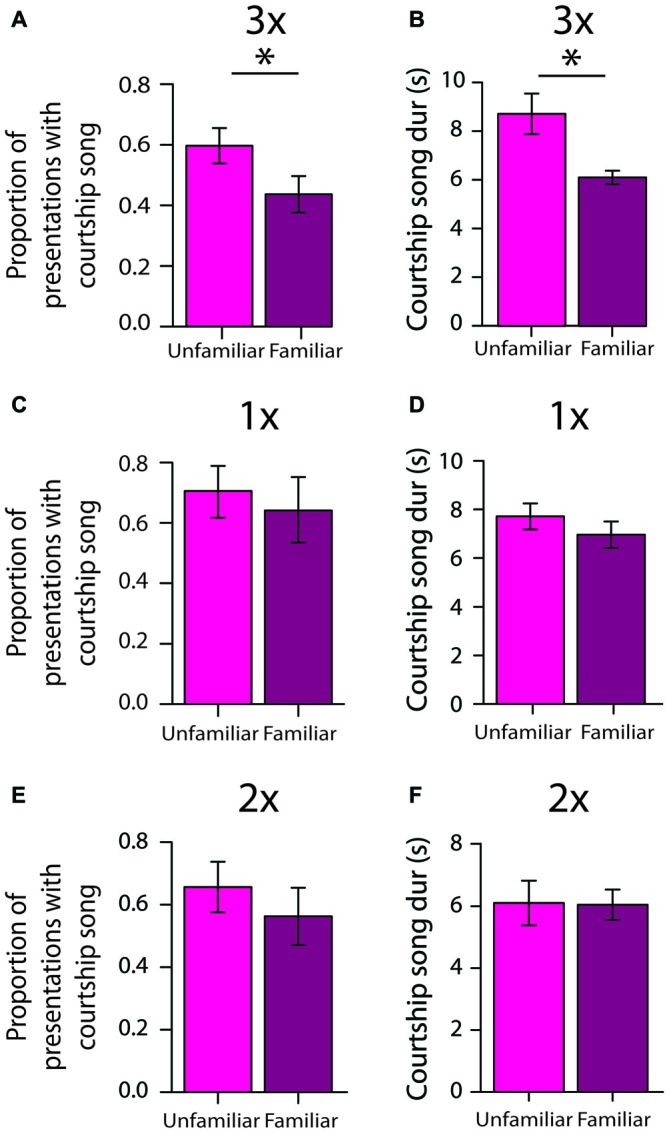
**Repeated exposures to females are important for the persistence of social memories over days (longer-term memory tests). (A)** When tested 2 days after 3X tests, male Bengalese finches were significantly less likely to produce courtship song to familiar females than to unfamiliar females (*n* = 13 males; Table 2). Plotted are the average (±SEM) proportions of presentations with courtship song. **(B)** Two days after 3X tests, males produced shorter courtship songs to familiar females than to unfamiliar females (*n* = 7 males; i.e., only males that produced at least three courtship songs to familiar females and at least three courtship songs to unfamiliar females). **(C)** Two days after 1X tests, males did not differentially produce courtship song to unfamiliar and familiar females (*n* = 7 males). **(D)** Two days after 1X tests, males produced courtship songs to familiar and to unfamiliar females that were of similar duration (*n* = 6 males; i.e., only males that produced at least three courtship songs to familiar females and at least three courtship songs to unfamiliar females). **(E)** Two days after 2X tests, males did not differentially produce courtship song to unfamiliar and familiar females (*n* = 10 males). **(F)** Two days after 2X tests, males produced courtship songs to familiar and to unfamiliar females that were of similar duration (*n* = 8 males; i.e., only males that produced at least three courtship songs to familiar females and at least three courtship songs to unfamiliar females). For all panels, “*” indicates *p* < 0.05.

To assess the persistence of social memories for individual females, we investigated courtship song production 2 days after initial exposures to females (e.g., 2 days after 3X tests; “longer-term memory tests”). We analyzed the degree to which males differentially produced courtship song to familiar females (i.e., females that males were exposed to 2 days earlier) and unfamiliar females (i.e., females that males were not exposed to 2 days earlier). Two days after 3X tests, male Bengalese finches (*n* = 13) were significantly less likely to produce courtship song to familiar females than to unfamiliar females (*F*_(1,12)_ = 7.0, *p* = 0.0213; Figure 5A; Table 2). On average, males produced courtship song to familiar females on only 41.0 ± 6.1% of presentations whereas they produced courtship song to unfamiliar females on 58.7 ± 6.1% of presentations. This difference could be due to a decrease in courtship song production towards familiar females or to an increase in courtship song production towards unfamiliar females; therefore, we compared a male’s courtship song production to familiar and unfamiliar females on longer-term tests against courtship song production during the first exposure to females during 3X tests. Males were significantly less likely to produce courtship song when they were exposed to familiar females during longer-term memory tests than when they were exposed to these same females for the first time during 3X tests (*F*_(1,12)_ = 20.3, *p* = 0.0007). Additionally, males were as likely to produce courtship song to an unfamiliar female during longer-term memory tests as to an unfamiliar female during the 3X tests (*F*_(1,12)_ = 0.0, *p* = 0.9448). Therefore, the difference in courtship song production during longer-term memory tests was due to a decrease in courtship song production to familiar females.

**Table 2:** **Number of stimulus females and number and proportion of presentations with courtship song for each male during longer-term memory tests 2 days after 3X tests**.

		UNFAMILIAR FEMALES		FAMILIAR FEMALES	
Male ID	# of females	# of presentations with courtship song	proportion of presentations with courtship song	# of presentations with courtship song	proportion of presentations with courtship song
o174br12	6	5	0.83	3	0.50
bl12w13	8	6	0.75	5	0.63
black	8	5	0.63	2	0.25
o189br53	8	2	0.25	3	0.38
s1408g	8	7	0.88	4	0.50
p168y48	8	4	0.50	3	0.38
p173y53	8	6	0.75	5	0.63
p183y25	8	5	0.63	2	0.25
bl27p163	8	3	0.38	1	0.13
bl93p188	8	3	0.38	4	0.50
p177y27	8	2	0.25	1	0.13
w34o156	8	5	0.63	7	0.88
o188br52	10	8	0.80	2	0.20

*For these tests, males were individually presented with unfamiliar and familiar females only once and were exposed to the same number of unfamiliar and familiar females*.

To assess the degree to which song performance could reflect longer-term memory for individual females, we compared various measures of song performance between courtship songs produced to familiar and to unfamiliar stimulus females 2 days after 3X tests. We analyzed songs only from males that produced at least three courtship songs to unfamiliar females and at least three courtship songs to familiar females (*n* = 7 males), and for each male we examined, on average, 5.1 ± 0.5 (range: 3–7) courtship songs directed at unfamiliar females and 4.6 ± 0.6 (range: 3–7) courtship songs produced to familiar females. Courtship song durations were shorter when males produced courtship songs to familiar females than when they produced courtship songs to unfamiliar females (*F*_(1,6)_ = 16.7, *p* = 0.0064; Figure 5B). The durations of courtship songs were shorter when males were exposed to familiar females during longer-term memory tests than when they were exposed to the same females for the first time during 3X tests (*F*_(1,6)_ = 17.6, *p* = 0.0057). Additionally, the durations of courtship songs that males produced to unfamiliar females during longer-term memory tests were comparable to the durations of courtship songs that males produced to unfamiliar females during the 3X tests (*F*_(1,6)_ = 3.52, *p* = 0.1099).

Song tempo (i.e., sequence durations), the number of introductory notes preceding song, the mean and variability of FF, and variability of syllable sequencing were not significantly different between courtship songs produced to familiar and to unfamiliar females (*p* > 0.20 for all analyses).

To gain insight into the amount of exposure that is required for the formation of longer-term social memories, we investigated whether males demonstrated similar differences in courtship song production after only one (*n* = 7 males) or two exposures (*n* = 10 males) to individual females. In contrast to the differential production of courtship song to unfamiliar females 2 days after 3X tests, males produced courtship song to familiar (64.5 ± 12.9%) and to unfamiliar (70.6 ± 10.0%) females to the same degree 2 days after 1X tests (*F*_(1,6)_ = 0.2, *p* = 0.6371; Figure 5C). The duration of courtship songs produced to familiar and to unfamiliar females was also not significantly different (*F*_(1,5)_ = 1.5, *p* = 0.2710; Figure 5D).

Similarly, male Bengalese finches did not differentially produce courtship song to unfamiliar and familiar females 2 days after 2X tests (*F*_(1,9)_ = 2.8, *p* = 0.1286; Figure 5E), and the durations of courtship songs produced to familiar and to unfamiliar females were not significantly different (*F*_(1,7)_ = 0.0, *p* = 0.8490; Figure 5F). This lack of longer-term memory was not due to a lack of a short-term memory formation. During 2X tests, these same males were significantly less likely to produce courtship song on the second exposure to an individual female than on their first exposure to the female 4–5 min earlier (*F*_(1,9)_ = 46.4, *p* < 0.0001), and the magnitude of this decrease was comparable to the magnitude observed during the 3X tests.

## Discussion

A hallmark of social memory is the differential display of behavior toward familiar and unfamiliar individuals. For example, across a variety of species, males spend less time investigating, attempt fewer copulations with, and vocalize less in response to familiar females (e.g., Dewsbury, 1981; Thor and Holloway, 1982; Johnston and Rasmussen, 1984; Bluthe et al., 1990; D’Amato and Moles, 2001; Lukas et al., 2013; Schnell et al., 2014). Individual recognition also modulates female mating decisions, as females preferentially mate with males that they have observed winning aggressive interactions or mating with other females (Mennill et al., 2002; Valone and Templeton, 2002; Galef, 2008). However, little is known about the rapidity with which social familiarity affects the display of courtship behaviors like birdsong, or the degree to which social memory differentially affects different aspects of courtship behavior.

Here, we used a habituation-dishabituation assay to investigate how familiarity affected the motivation to produce courtship song and the performance of courtship song in the Bengalese finch. We document that a single, brief exposure to a female (<30 s) decreased the likelihood that a male would produce courtship song upon re-exposure to that same female 5 min later (i.e., habituation) and that exposing a male to a different female after repeated exposures to the same female increased the likelihood of courtship song (i.e., dishabituation). We argue that such decreases in courtship song following re-exposure to females reflect the formation of social memories for individual females and do not solely reflect fatigue on the part of the male. This is because the proportion of males producing courtship song decreased at a faster rate when males were exposed to the same female across consecutive presentations than when males were exposed to different females across consecutive presentations. Further, 2 days after repeated exposures to individual females, males remained less likely to produce courtship song to familiar females than to unfamiliar females, suggesting the formation of longer-term social memories. This longer-term memory for females seemed to require at least three consecutive exposures to a female, since the differential production of courtship song to unfamiliar females was observed after 3X tests but not after 1X or 2X tests. However, in contrast to the influence of familiarity on the motivation to produce courtship song, various measures of song performance (e.g., song tempo, syllable structure and sequencing) were not affected by social memory formation. These data reveal the persistence and rapid formation of social memories and the distinct effects of familiarity on motivational and performance aspects of courtship song.

The decrease in the production of courtship song to individual females could not only signify the formation of social memories for individual females but also represent an adaptive change in male behavior. Female responses during social interactions have been found to influence male courtship and vocal behavior in a variety of ways. For example, feedback from females modulates the intensity of courtship displays in bowerbirds and the trajectory of vocal learning in juvenile male cowbirds (West and King, 1988; Patricelli et al., 2002; Miller et al., 2008; Dohme et al., 2015). Furthermore, “rejection” behaviors by females or a lack of positive reinforcement following exposure to females can lead to a decrease in the expression of courtship or appetitive behaviors (e.g., Griffith and Ejima, 2009; Cornil and Ball, 2010). In our experiments, male Bengalese finches were denied the opportunity to copulate with stimulus females, and it is possible that males interpreted that lack of successful mating following courtship song as rejection. Consequently, the decrease in the production of courtship song could represent an adaptive response to divert reproductive effort away from females that will not allow mating opportunities. Further, while we did not notice conspicuous changes to the behavior of stimulus females across presentations, it is possible that stimulus females’ behavior toward males subtly changed across repeated exposures in ways that could have influenced a male’s motivation to produce courtship song.

In contrast to the rapid effects of familiarity on the motivation to produce courtship song, familiarity did not significantly influence the performance of song features that can influence the attractiveness of a male’s song, including song tempo, syllable structure, or syllable sequencing (Catchpole and Slater, 2008; Sakata and Vehrencamp, 2012; Dunning et al., 2014). However, our data do not rule out the possibility that social familiarity can affect song performance. Indeed, a previous study found that the sequencing of syllables during courtship song becomes more variable (i.e., more like non-courtship song) across longer periods of exposure to females (Heinig et al., 2014). The authors housed male Bengalese finches next to individual females for one hour and found that syllable sequencing was more stereotyped for the first three courtship songs than for courtship songs produced during the remainder of the hour. The first three courtship songs were produced within minutes after the introduction of the female, whereas the courtship songs produced later in the testing period were produced, on average, over 30 min after the introduction of the female. In our experiment, we only briefly exposed males to females (<30 s per presentation) and generally contrasted a male’s song performance during his first exposure to a female to his song performance during subsequent exposures within 10 min of the first exposure. Together, these studies suggest a hierarchy of influences on courtship song and that social familiarity affects the motivation to sing more rapidly than it affects the performance of song.

The circuit connecting the medial preoptic area (POM), midbrain areas like the ventral tegmental area (VTA) and periaqueductal gray (PAG), and song control nuclei is hypothesized to influence various aspects of song production, including the motivation to sing (Riters and Ball, 1999; Goodson, 2005; Bharati and Goodson, 2006; Yanagihara and Hessler, 2006; reviewed in Balthazart and Ball, 2007; Alger et al., 2009; Goodson et al., 2009; Riters, 2012). In particular, dopaminergic projections from the VTA and PAG are thought to influence the motivation to produce courtship song. This idea is supported by findings that dopaminergic neurons in these areas are more active during the production of courtship song than during the production of non-courtship song (Yanagihara and Hessler, 2006; Hara et al., 2007; Goodson et al., 2009; Matheson and Sakata, 2015a). However, dopamine from the VTA and PAG has also been found to affect the performance of song (reviewed in Woolley and Kao, 2015). Dopamine concentrations in song control nuclei such as the basal ganglia nucleus Area X are elevated when male songbirds produce courtship song (Sasaki et al., 2006; Ihle et al., 2015), and pharmacological manipulations that increase dopamine concentrations in the brain increase the stereotypy of syllable structure and sequencing (Matheson and Sakata, 2015a). Further, antagonism of dopamine receptors in Area X inhibits the social modulation of song structure (Leblois et al., 2010; Murugan et al., 2013). Consequently, if changes to VTA and PAG activity are central to the decrease in courtship song production following repeated exposures to females, we should similarly observe changes to various measures of song performance across repeated exposures to females. Because we did not observe changes to song performance, our findings suggest that mechanisms independent of dopamine could also contribute to changes to courtship song production that accompany social memory formation. For example, because arginine vasotocin (AVT; the non-mammalian homolog of vasopressin) influences the motivation to produce courtship vocalizations without affecting acoustic structure in species like the plainfin midshipman (e.g., Goodson and Bass, 2000, 2001; Goodson and Thompson, 2010), AVT could mediate the observed changes to courtship song production in male Bengalese finches.

Regardless of the precise neurochemical mechanisms underlying the effects of familiarity on the production of courtship song, our data support the notion that motivational and performance aspects of social behaviors can be dissociated (Riters and Balthazart, 1998; reviewed in Balthazart and Ball, 2007; Cornil and Ball, 2010). Our findings that repeated exposures to individual females affected the motivation to produce courtship song without affecting various measures of song performance bear striking similarity to the effects of testosterone in the POM on singing behavior in canaries (Alward et al., 2013). In canaries, testosterone implants directly into the POM increase the prevalence and duration of song but do not affect song stereotypy. Despite that testosterone exerts its effects in the POM over days whereas familiarity rapidly modulates song production over minutes, these behavioral similarities suggest that the mechanisms through which social memory affects song production in Bengalese finches could resemble the mechanisms through which testosterone in the POM affects song production in male canaries.

Taken together, our studies reveal the ability of male songbirds to rapidly form social memories of females, the importance of repeated exposures for the longer-term persistence of social memories, and the dissociable effects of familiarity on motivation and performance. These data highlight the role of cognitive processes in social behavioral expression and motivate further investigations into the neurochemical systems that underlie the formation of short- and longer-term social memories.

## Author Contributions

DCT helped design experiments, collect and analyze data, write manuscript. HS helped design experiments, analyze data, write manuscript. JTS helped design experiments, analyze data, write manuscripts.

## Conflict of Interest Statement

The authors declare that the research was conducted in the absence of any commercial or financial relationships that could be construed as a potential conflict of interest.
